# Representative Volume Element (RVE) Analysis for Mechanical Characterization of Fused Deposition Modeled Components

**DOI:** 10.3390/polym13203555

**Published:** 2021-10-15

**Authors:** Patrich Ferretti, Gian Maria Santi, Christian Leon-Cardenas, Elena Fusari, Giampiero Donnici, Leonardo Frizziero

**Affiliations:** Department of Industrial Engineering, Alma Mater Studiorum, University of Bologna, I-40136 Bologna, Italy; patrich.ferretti2@unibo.it (P.F.); gianmaria.santi2@unibo.it (G.M.S.); elena.fusari2@studio.unibo.it (E.F.); giampiero.donnici@unibo.it (G.D.)

**Keywords:** FEM, FDM, additive manufacturing, microstructure behavior, linear analysis, RVE

## Abstract

Additive manufacturing processes have evolved considerably in the past years, growing into a wide range of products through the use of different materials depending on its application sectors. Nevertheless, the fused deposition modelling (FDM) technique has proven to be an economically feasible process turning additive manufacture technologies from consumer production into a mainstream manufacturing technique. Current advances in the finite element method (FEM) and the computer-aided engineering (CAE) technology are unable to study three-dimensional (3D) printed models, since the final result is highly dependent on processing and environment parameters. Because of that, an in-depth understanding of the printed geometrical mesostructure is needed to extend FEM applications. This study aims to generate a homogeneous structural element that accurately represents the behavior of FDM-processed materials, by means of a representative volume element (RVE). The homogenization summarizes the main mechanical characteristics of the actual 3D printed structure, opening new analysis and optimization procedures. Moreover, the linear RVE results can be used to further analyze the in-deep behavior of the FDM unit cell. Therefore, industries could perform a feasible engineering analysis of the final printed elements, allowing the FDM technology to become a mainstream, low-cost manufacturing process in the near future.

## 1. Introduction

The process of three-dimensional (3D) printing, known as additive manufacturing (AM) has achieved an unexpected evolution. The ability of producing most kinds of complex, irregularly shaped geometries is an asset for this technology. Moreover, the rapid increase in design software makes 3D printing ideal for manufacturing custom components impossible to be produced at the industrial level by using standard processes. Nowadays, many AM technologies for polymers offer high levels of material and aesthetics quality such as stereolithography (SLA), selective laser sintering (SLS), digital light processing (DLP), and ink-deposition technologies including the Polyjet (patended by Stratasys [1,2,3,4,5]. In contrast, these methodologies may result expensive due to the uniqueness of each process, materials availability, and the need of a more expensive, specialized equipment [6].

Fortunately, the fused deposition modelling (FDM) technology have evolved substantially over the last years, with the arrival of a wide range of filaments and materials, leading to higher manufacturability and aesthetic quality. In addition, subsequent advances on printing machines have led to multi-material deposition and finer surface quality. Software also offers a wide range of possibilities from open sources to commercial solutions underlying the interest in G-code optimization. To catch the opportunity of making FDM available for industrial applications, it is necessary to deeply understand and predict the behavior of printed components. The derived mechanical tensile response and the elongation percentage of the part would help designers to close an important gap to product industrialization. The studies currently available in the literature present limited results or focus on a single component and are difficult to be extended, such as the study of Somireddy et al. that considered the behavior of a foil section [7]. Although the proposed model is built on an image of a printed section, it does not allow reducing the size of voids and therefore a variable model was not obtained. Furthermore, the tests conducted used beam elements, but the number of elements was enormously high, and it was a difficult approach to apply on large components. The model presented by Bhandari et al. is very interesting, because it divided the model into two well-defined areas, i.e., the infill and the contour lines [8]. The infill is schematized as a series of beams, and the contour act as shell elements. Again, it is difficult to extend the model to complex components, but it is very interesting from a mathematical point of view. The study of Garg and Bhattacharya proposes a methodology that faithfully reproduces the layer and line deposition typical of the FDM-printed model [9]. However, it is extremely computationally demanding to mesh each individual line with enough elements. Following this approach, even the simplest geometry requires extremely high computing power and time. This proposal builds a model based on the image analysis of the mesostructure of the molded component. In this way, the representative volume element (RVE) model is not fixed in its dimensions, but every time the material or printing parameters are changed based on the image analysis. Thanks to the RVE model, it is then possible to use macroelements that summarize the mesoscopic properties of the component and allow complex components to be studied with relative ease.

### 1.1. FDM Part Micromechanics

It is known that AM is a technology in which its understanding relates to the high variability of the process and environment characteristics. This variability can be translated into correct parameter definition issues, which otherwise would lead to an internal anisotropy, making this type of resulting material very complex to analyze. Extruded material irregularities due to FDM processes are discussed previously by Lee et al. [10] and Kotlinski et al. [11], which agree that anisotropy leads to limitations in obtaining feasible prototype properties. Currently, only a few studies currently have started to analyze the internal behavior of FDM-produced parts. An FEM modelling of the mesostructure of FDM-printed parts was discussed by Somireddy et al. [12]. The FEM analysis was used to find the elastic modulus of a single printed layer of a unidirectional (UD), polymer-extruded material. The authors have laid down an understanding in order to gather an FEM analysis that took into account the anisotropic behavior of the 3D printed part. Additionally, the study of Bhandari and Lopez-Anido underlies a distinctive, rather interesting approach to material anisotropy [8], as the FEM analysis was performed in a lattice-like internal structure, with an about 20% difference in Poisson’s ratios with respect to the test values.

Nevertheless, the accurate analysis of the mesostructure of the printed material is therefore needed to gather an FEM methodology that could allow the analysis of whole structures manufactured by FDM. By understanding the variability characteristics of the printed structure in a single, homogeneous element helps to further study the part’s macroscopical behavior. The analysis of composites structures as an unique element in FEM could assess accurately changes by any of their components, being able to differentiate different resins analogous to those in the study by Croccolo et al. [13]. A second study by Somireddy et al. established a numerical homogenization procedure for a more efficient material modeling of the printed parts [14]. This model claimed to gather the influence of printing variables such as build orientation, printing direction, and layer thickness. A layer deposition influence is noted as well, and it has been stated that the material responses to different parts of the modelled structure are dependent on the build orientations and thicknesses of the parts.

### 1.2. RVE FEM Analysis

Computational analysis tools have given engineers the capacity of create new methodologies able to enhance the understanding of physical phenomena in modern, multimaterial, anisotropic problems. The state-of-the-art developments of complex structures have evolved thanks to the introduction of composite materials. Nowadays, some analysis of yield and failure theories in polymer–matrix composites (PMCs) are possible [15]. This is due to a unified theory that can be used to predict their nonlinearity and strength by also considering the anisotropy and tension-compression asymmetry simultaneously. Another one-parameter yield function was proposed earlier by Sun and Chen to establish a nonlinear plastic model for UD PMCs [16,17]. Additionally, Mellinger et al. described polymer–air composites, and they showed that they are elastically softer due to the air content and in relation to the size and shape of the polymer walls [18]. This behavior defines foam structures as described by Ashby in [19]. Because of these considerations, the influence of air is relevant with respect to its volume, and it should be considered for FDM-made component simulations. Using FEM software is indeed possible to calculate mechanical behaviors of anisotropic materials, i.e., composites, using a micromechanical RVE [20]. RVE is the smallest volume in which a measurement can be made in order to homogenize the entire domain. In the studied case, a periodic unit cell is a simpler choice. ANSYS software elaborates the area of analysis by means of the material cross-section geometry. The RVE study of Bhaskara et al. started from periodic boundary conditions, applied to the RVE to calculate elastic modulus of composite elements, which are characterized as an anisotropic behavior [21]. Most micromechanical models are applied to a composition of fibers immersed in a matrix, so that an RVE or unit cell can be insulated. This methodology is used to study composite materials (e.g., UD carbon fibers in an epoxy matrix) and anisotropic materials in general. Because of the similarity between long-fiber RVE and FDM deposition, the authors choose this approach to analyze the resulting mesostructure of a 3D printed component. Printed lines are similar to UD carbon fibers composites. The voids between lines resemble the structure of porous materials such as foams. FDM-printed parts are in fact made of air and polymeric materials and can be considered “composites”.

The RVE methodology has proven to show good results for the analysis of anisotropic materials.

## 2. Materials and Methods

### 2.1. Microstructure Definition

It is necessary to define the minimum volume to be analyzed by means of the RVE. Because of the previous literature findings and the study performed by Grimal et al. [22], it is possible to state that the structure of a 3D printed material can be defined as periodic. The aim of this research was to create a methodology to study the behavior of 3D printed components. This model would start from a linear, discrete model and then be extended to a model that can consider diverse material and process variabilities that deliver such nonlinearities to polymeric materials due to the 3D printing process. Furthermore, to guarantee the continuity between both models, it was decided to use a unit cell with an actual size slightly greater than that of a single line. However, Section 3.1 highlights this aspect in the linear field. Considering overlapping lines revealed no loss or addition of information by varying the size of the RVE.

### 2.2. Model Considerations

**Figure 1:**
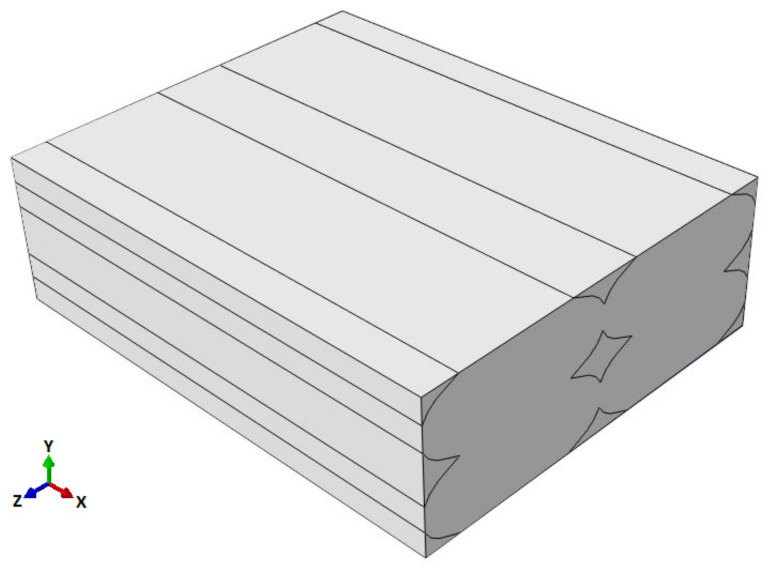
The representative volume element (RVE) and the reference system.

- The material used to validate this model was polylactic acid (PLA). The average material values used for the Young’s modulus, Poisson’s ratio, and density came from specimens made through injection molding [23,24,25].
- The stress–strain behavior of the material was approximated as linear. This allowed simplifying the problem but limits the validity of the model to small deformations, while generally the behavior of polymers is nonlinear.
- The model does not take into consideration that there is a preferential orientation of the polymer chains along the direction of extrusion during the 3D printing process. [26]. The value of the mesoscopic geometry plays a key role, given the fact that such geometry defines the anisotropy of the structure. Different geometry schematizations led to different results from both a numerical and a physical point of view.
- The condition of “perfect bonding” was set between various layers, so the assumptions that the adhesion is perfect at the interface between one layer and the next and between one line and the adjacent one was taken. This hypothesis allowed keeping the model linear, avoiding problems of nonliner contact behavior. Due to this hypothesis, the results reported higher values for *E*_22_ and *E*_33_ (Young’s moduli along directions perpendicular to the fiber direction) and the shear modules, according to the reference system, as seen in Figure 1. However, *E*_11_ (Young’s modulus along the “fiber” direction) should remain unchanged because it is not affected by the layers’ adhesion.

However, the possibility of an optimization of the microstructure in order to reduce voids, as previously presented in [27], was not taken into consideration. This is due to the hypotheses considered in this study beforehand that established a perfect bonding with no lines or layer boundaries. An optimization process will lead to no appreciable result.

The specimens were made with an E3D Tool-changer 3D printer, and the toolpath (G-Code) was obtained using Cura 4.9.1 (Ultrecht, The Netherlands) free slicing software.

The main printing parameters are reported in the Table 1; it should be noted that different printers, with the same type of material but from a different supplier (brand), would give different results in terms of microstructure. An image analysis on the specimen failure section is always necessary to evaluate the obtained geometry and therefore to remodel the RVE accordingly. To sum up, different volume-area ratios of voids produce different behaviors in various directions.

### 2.3. Additional Image Analysis Considerations

**Figure 2:**
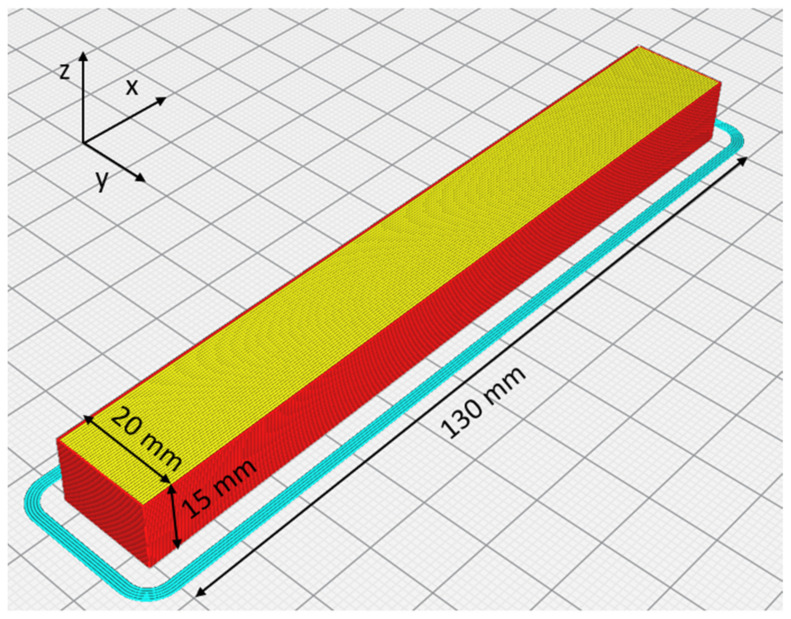
Dimensions of the specimen at the end of the slicing process in Cura.

The specimen microstructure was obtained using an optical microscope with a 20× magnification. The specimen must be long enough to allow the printing speed and extrusion rate to stabilize. To simplify the geometry of the RVE model, the specimen must be printed with lines parallel to the largest dimension of the specimen, as shown in Figure 2, in order to be able to clearly and uniquely highlight the microstructure, once broken at 90° with respect to the largest dimension. The choice of creating a parallel line RVE model is dependent by the following reasons:

- Overlapping parallel lines are always found on the contour (perimeter) of the component. An example is thinner wall parts in general; in this case, the walls often form the entire structure of the printed component. This is not the conventional way of 3d printing, but it is still significant for certain type of parts.
- On tests carried out on FDM-printed components, where bending and torsional loads were predominant, compared to tensile loads, it was observed by the authors that the initialization and propagation of cracks started from the external surface of the component. This is a further incentive to create an RVE model with parallel lines to understand better the behavior in that location.
- The presented model is linear with a constraint of the perfect adhesion between layers, leading to a simplified model for tensile tests. In any case, it is possible to create a 45°–45° RVE model by using the same methodology but needs the study of a new RVE model.

When the printer started extruding a new line, its speed started from zero and grew up to the cruise value in accordance with the acceleration set in the Firmware or directly in the slicing software.

Furthermore, the extruded material flow could vary in areas where the speed was not constant, changing the volume/area ratio of the voids. There are effective solutions to adjust the material flow rate during the acceleration and deceleration phases, for example “coast at end” and “retraction extra prime amount” in the slicing software or even directly in the Firmware such as “Lin advance” in Marlin firmware. The possibility of a nonperfect matching between the general and the local microstructures in these areas may still exist.

The specimen must be broken in a brittle manner, and the failure must be carried out in the central area of the sample. In many cases, the need for a heated bed platform is essential to ensure adhesion to the build plate.

**Figure 3:**
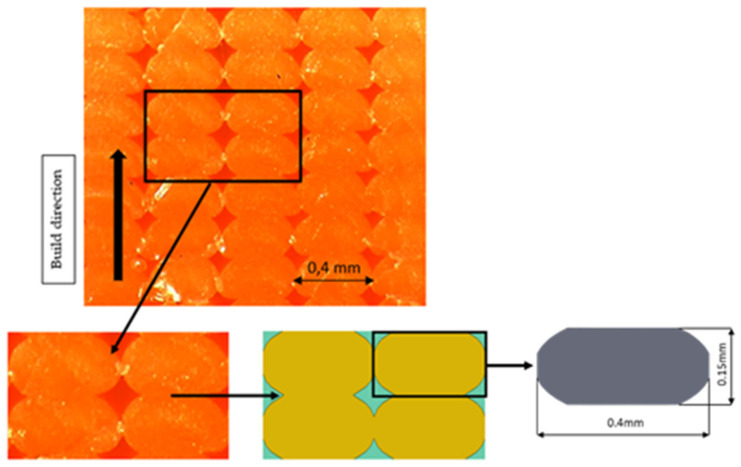
Workflow of geometry selection.

The height of the first layer was extremely dependent on the calibration of the distance between the nozzle and the build plate. The combination of these two aforementioned factors can produce a series of layers with variations in microstructure. The image necessary to create the RVE model must therefore be taken in the medium–high parts of the specimen, as shown in Figure 3, to avoid influences on the height of the first layer and the temperature of the printing surface.

### 2.4. Input Data

The geometry used was similar to a UD-fiber composite RVE and was therefore composed by PLA and “Air”. The second one is necessary for the definition of the elementary volume which needs to have parallel plane faces with the same number of nodes on the opposite surfaces. This type of mesh is called periodic mesh. The reference values for these materials are given in Table 2.

Moreover, the values of the “Air” material were assumed from the Ansys database, and they had no structural meaning in the simulation. The influence of this material on the mechanical behavior of the structure was in fact irrelevant. It could have a nonzero influence in the case of the thermal analysis of cooling or heating of the component being air as an insulating material. However, this analysis is beyond the scope of this study, and therefore for the simplicity and clarity, the “air” material was considered “linear elastic” to maintain compatibility in the simulation. The air material results, however, were mandatory to perform the RVE analysis, since the calculation of the various modules was weighed on the lateral area of the RVE. Therefore, for the simplicity of calculation of the software, it is a good practice to keep a parallelepipedal shape.

Moreover, since it is currently a linear model, it is only valid for small deformations. However, if a large deformations analysis is performed with nonlinearities or thermal analyses, the contribution of air would be crucial for validating the idea of implementing air in the system.

### 2.5. Geometry

**Figure 4:**
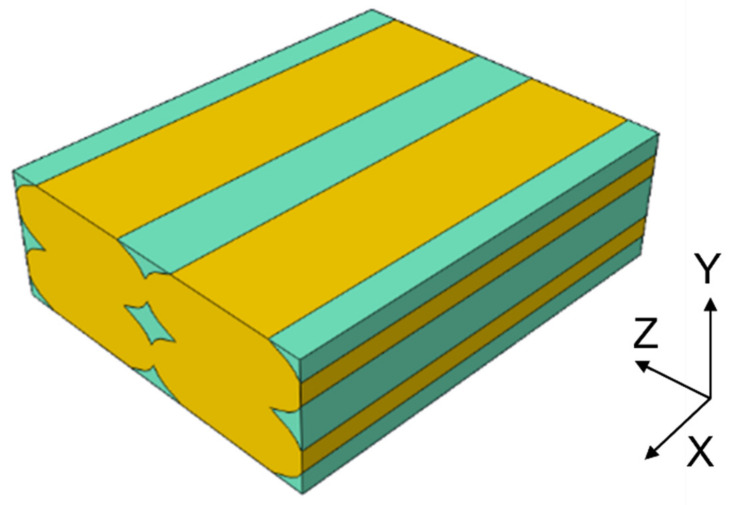
RVE model. The polylactic acid (PLA) mesostructure is indicated in yellow, and air is indicated in green.

The geometry followed the observations under the microscope. The dimensions were obtained by analyzing the section image of the specimen. It was decided to consider a single unit of length for the “Z” direction. The schematization of the printed lines was made considering an ellipsoidal section, while the contact areas were defined as continuous between adjacent lines and adjacent layers. Figure 4 shows that four lines were chosen in the definition of the RVE.

### 2.6. Loads and Constraints

**Figure 5:**
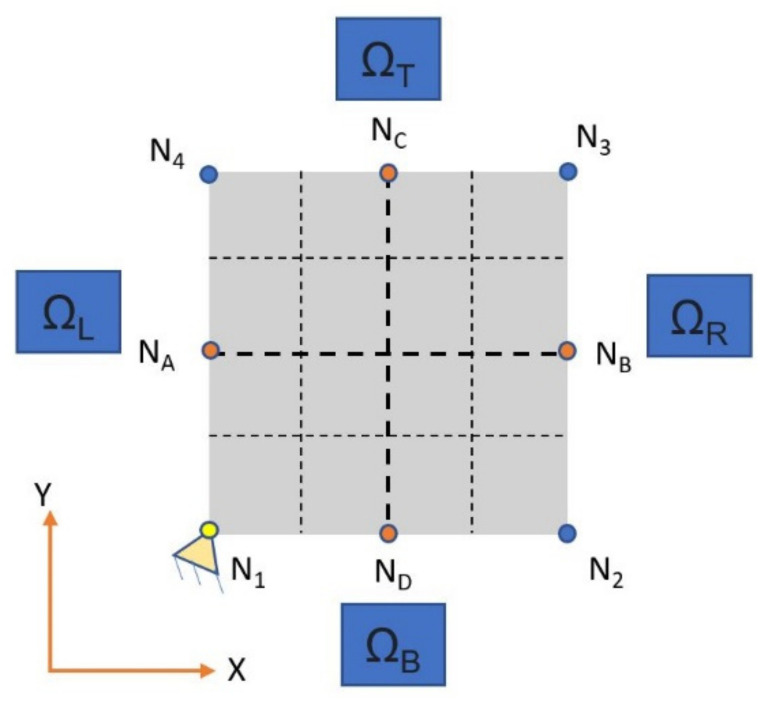
Two-dimensional (2D) RVE model for the application of boundary conditions.

In order to evaluate the characteristic modules of the homogenized material, it is necessary to apply particular boundary conditions to the fundamental volume. Specifically, Neumann conditions were not applied to the structure, but only Dirichlet conditions. Since the elementary volume was a part of the total volume, its behavior will be symmetrical with respect to the opposite surfaces. This involves the application of periodic boundary conditions to the model studied [28]. To clarify this, equations of the two-dimensional (2D) version are reported as Equation (1). The obtained 3D version of Figure 5 is an extension of these conditions. The displacement of the N-th node in the X and Y directions, $U_{\left(x,y\right)\;}^{N_{*}}$, were defined as follows:

(1) $$U_{\left(x,y\right)\;}^{N_{B}}-{\;U}_{\left(x,y\right)}^{N_{A}}-U_{\left(x,y\right)}^{N_{2}}+U_{\left(x,y\right)}^{N_{1}}=0\;U_{\left(x,y\right)\;}^{N_{C}}-{\;U}_{\left(x,y\right)}^{N_{D}}-U_{\left(x,y\right)}^{N_{4}}+U_{\left(x,y\right)}^{N_{1}}=0$$

**Figure 6:**
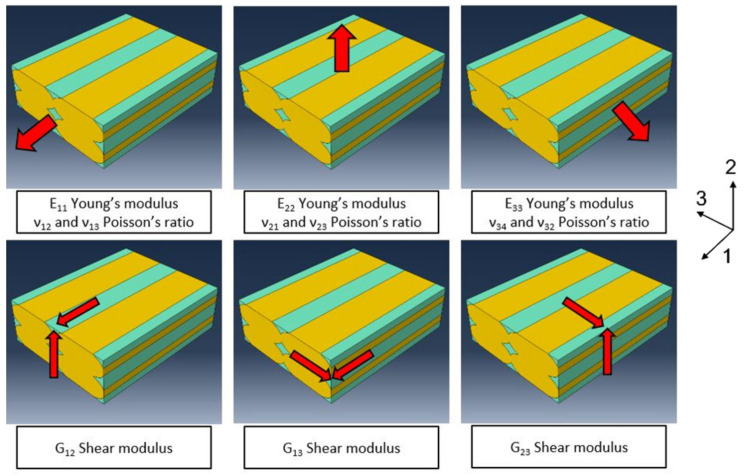
Load cases in the RVE model. The red arrows indicate the directions of the imposed displacement on the RVE. Software axes 1, 2, and 3 indicate the X, Y, and Z directions.

**Figure 7:**
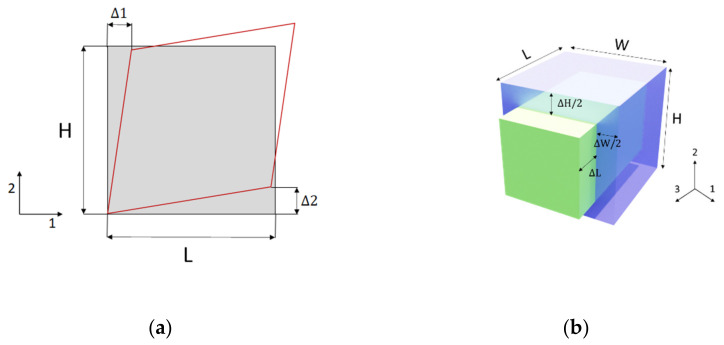
(**a**) Deformed part schematization of displacement to evaluate *G*_12_; (**b**) 3D schematization of the load case to evaluate *E*_11_ and Poisson’s ratios *ν*_12_ and *ν*_13_.

Subsequently, in order to find out the mechanical characteristics, particular conditions of the imposed displacement were studied afterwards. Figure 6 represents a schematization of various load cases. In Figure 7, there is the detailed 2D schematization of how the various shear moduli were calculated. Taking the G12 case and element characterization as examples, Young’s modulus *E*, Poisson’s ratio ν, and Shear modulus *G* were calculated according to [29]:

(2) $$E=\frac{Stress}{Axial\;strain}$$

(3) $$\nu =\frac{-Transvers\;strain}{Axial\;strain}$$

(4) $$E_{11\;}=\frac{\frac{\sum Front\;surface\;nodal\;forces_{in\;1-Direction}}{Front\;surface\;area\;\left(H\times W\right)}}{\frac{\Delta L}{L}}$$

(5) $$G_{12}=\frac{\frac{\sum Top\;surface\;nodal\;forces{\;}_{in\;1-Direction}}{Top\;surface\;area\;\left(L\times W\right)}}{\frac{\Delta 1}{H}+\frac{\Delta 2}{L}}$$

(6) $$G=\frac{Shear\;stress\;}{Tensors\;of\;shear\;strain}$$

(7) $$\nu_{12}=\frac{\frac{\Delta H}{H}}{\frac{\Delta L}{L}}$$

(8) $$\nu_{13}=\frac{\frac{\Delta W}{W}}{\frac{\Delta L}{L}}$$

## 3. Results

The obtained simulation results defined in the precedent chapter are displayed in Table 3.

**Figure 8:**
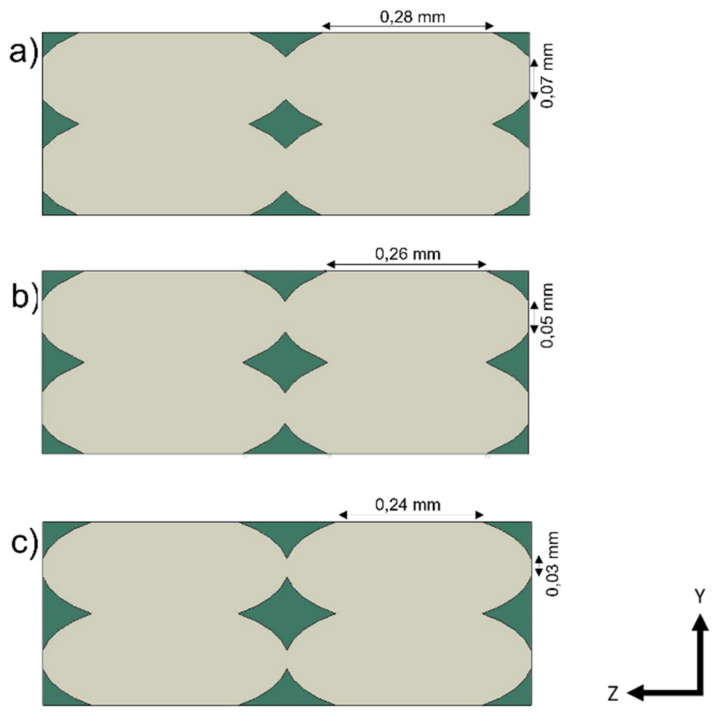
Increased RVE void length dimensions in Y and Z directions, respectively: (**a**) lengths of 0.07 and 0.28 mm; (**b**) lengths of 0.05 and 0.26 mm; (**c**) lengths of 0.03 and 0.24 mm.

As expected, there was a decrease in values in all directions due to the presence of voids in the structure. As for E_11_, its value was closely related to the resistant area, regardless of the geometry drawn. Along the direction of the lines, Young’ s modulus was in fact the highest, similar to the behavior of UD composites. The *E*_22_ and *E*_33_ responses depended on the contact area between adjacent lines and layers. By varying the printing parameters, it is possible to modify the geometry and its modules in the Y and Z directions. In the presented case study, the RVE model was linear, and there was a constraint of perfect adhesion between one layer and the next, i.e., continuity between the material of one line and the adjacent and overlying one. There would be a rather linear increase in the various moduli (Young’s and shear moduli) by varying the size of the voids and therefore increasing the contact area between the lines, until there were no more voids in the structure. Three kinds of RVE with different void dimensions were considered and tested, as shown in Figure 8. The same dimension of the RVE and the basic dimensions of the lines were maintained as reported in Figure 3. An increase of the contact area between the printed lines was obtained by keeping the same dimension of the RVE and reducing the voids dimension.

**Figure 9:**
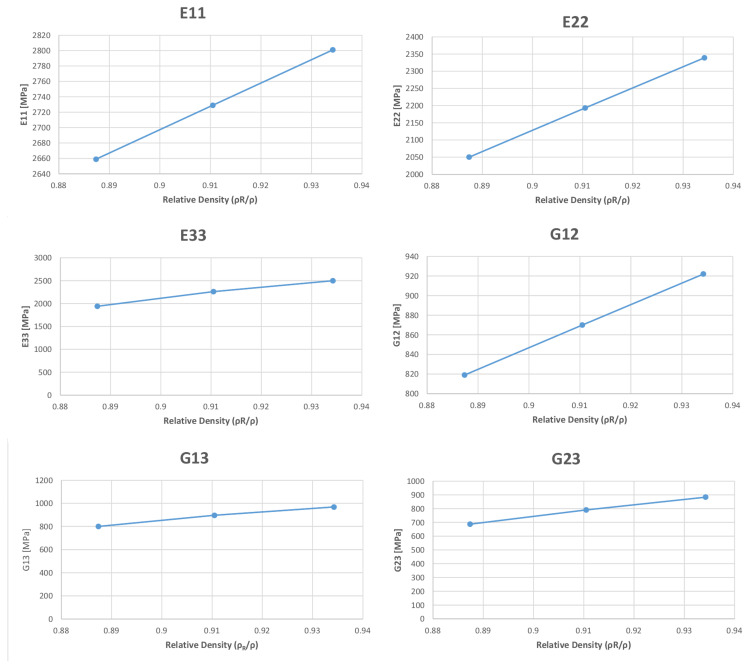
Variations of Young’s modulus and the Shear modulus with respect to the relative density.

Such reductions could be demonstrated by using the methodology proposed by Patrich et al. [27]. However, the variations of Young’s modulus and the Shear modulus values with respect to the relative density could be seen from the graphs reported in Figure 9. The data are shown in a similar manner as the reported research on a foam-like material by Imwinkelried [30] and Goods et al. [31], as the values increased in a linear way.

**Figure 10:**
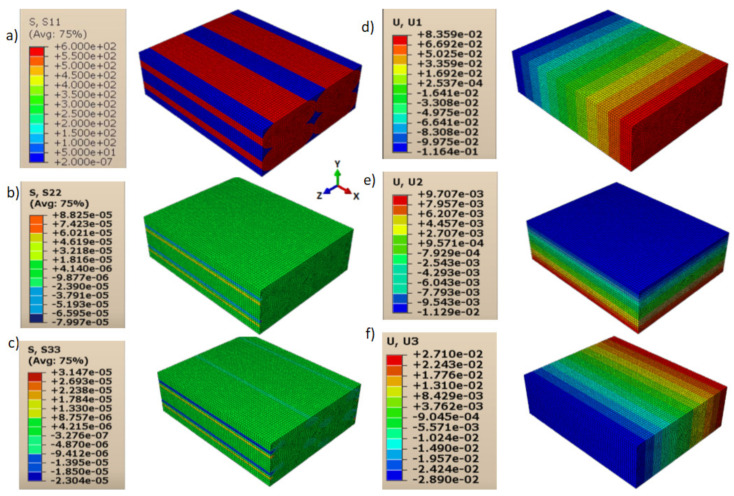
Load cases to evaluate *E*_11_: (**a**) stress in direction 11; (**b**) stress in direction 22; (**c**) stress in direction 33; (**d**) displacement in direction 11; (**e**) displacement in direction 22; and (**f**) displacement in direction 33.

**Figure 11:**
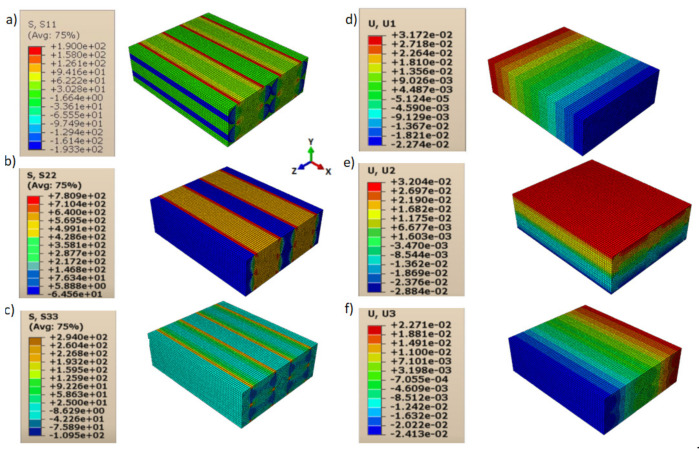
Load cases to evaluate *E*_22_: (**a**) stress in direction 11; (**b**) stress in direction 22; (**c**) stress in direction 33; (**d**) displacement in direction 11; (**e**) displacement in direction 22; (**f**) displacement in direction 33.

**Figure 12:**
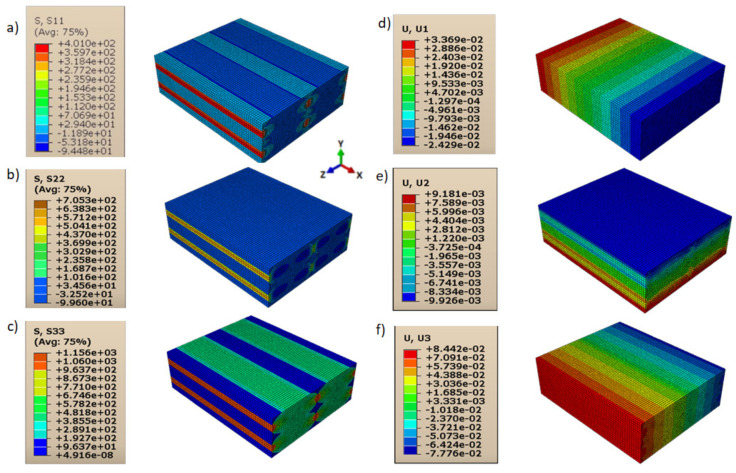
Load cases to evaluate *E*_33_: (**a**) stress in direction 11; (**b**) stress in direction 22; (**c**) stress in direction 33; (**d**) displacement in direction 11; (**e**) displacement in direction 22; (**f**) displacement in direction 33.

**Figure 13:**
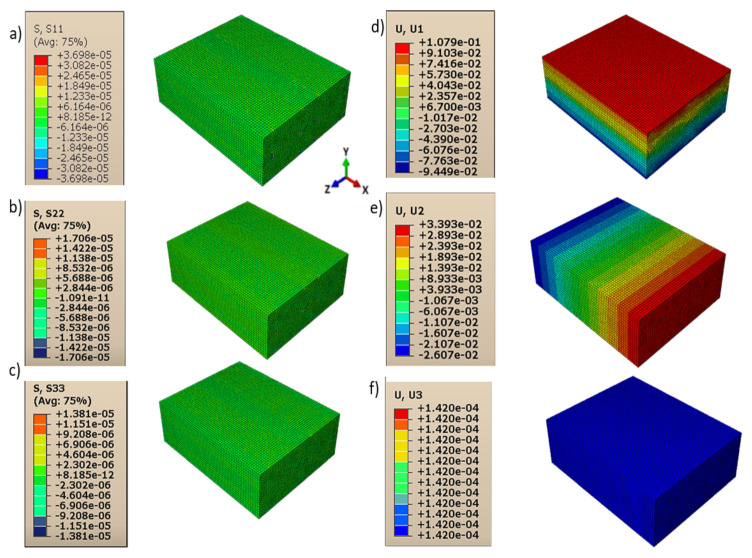
Load cases to evaluate *G*_12_: (**a**) stress in direction 11; (**b**) stress in direction 22; (**c**) stress in direction 33; (**d**) displacement in direction 11; (**e**) displacement in direction 22; (**f**) displacement in direction 33.

**Figure 14:**
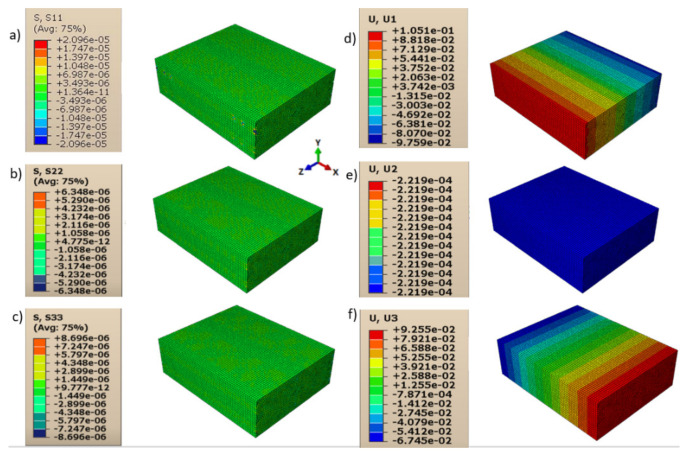
Load cases to evaluate *G*_13_: (**a**) stress in direction 11; (**b**) stress in direction 22; (**c**) stress in direction 33; (**d**) displacement in direction 11; (**e**) displacement in direction 22; (**f**) displacement in direction 33.

**Figure 15:**
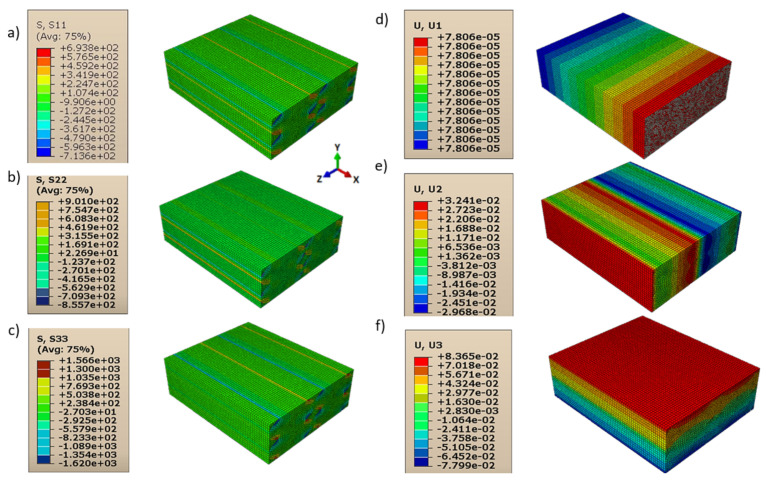
Load cases to evaluate *G*_23_: (**a**) stress in direction 11; (**b**) stress in direction 22; (**c**) stress in direction 33; (**d**) displacement in direction 11; (**e**) displacement in direction 22; (**f**) displacement in direction 33.

Consequently, shear moduli *G*_12_, *G*_13_, and *G*_23_ were reduced compared to those in the case of a homogeneous isotropic material and strictly depended on the contact behavior between layers and lines. The displacements and directional stresses of all the load cases to show the symmetric behavior of the RVE are reported in Figure 10, Figure 11, Figure 12, Figure 13, Figure 14 and Figure 15.

**Figure 16:**
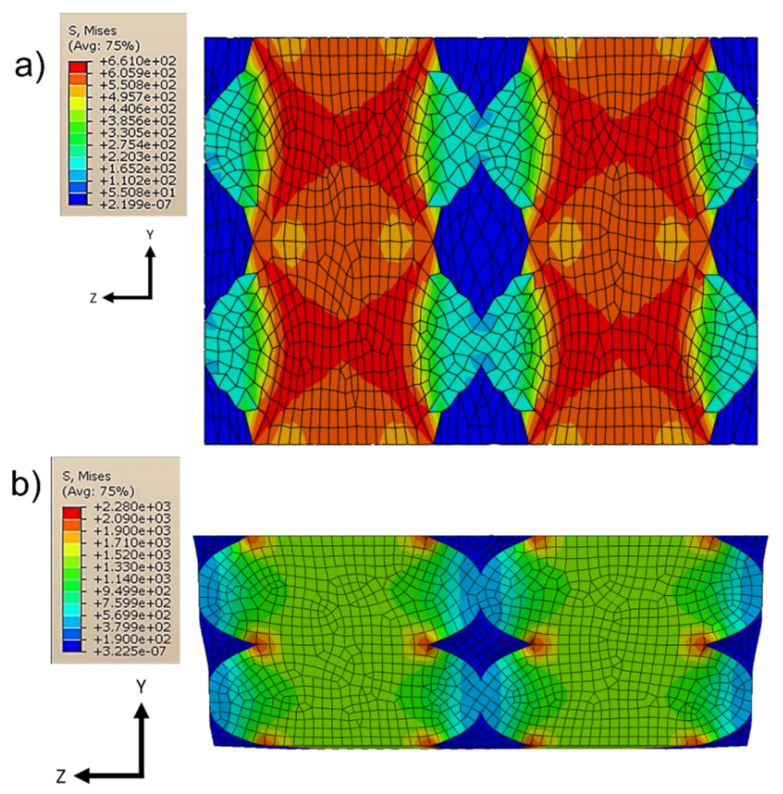
Von Mises stresses for *E*_22_ (**a**) and *G*_12_ (**b**).

**Figure 17:**
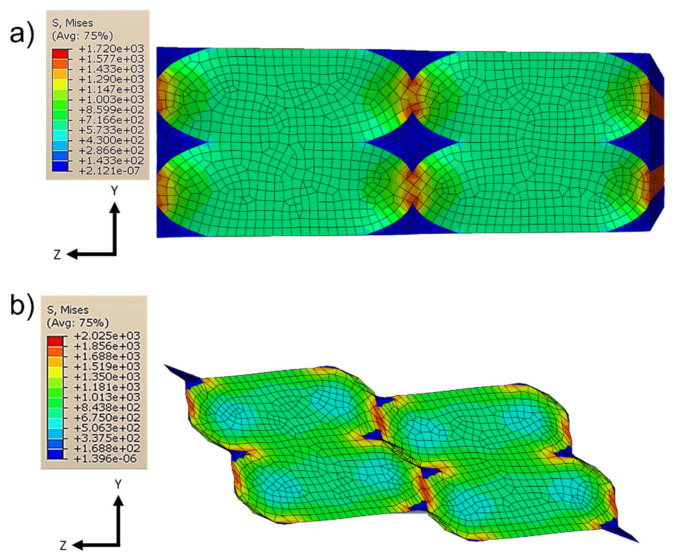
Von Mises stresses for *G*_13_ (**a**) and *G*_23_ (**b**).

It can also be noted in Figure 16 and Figure 17 that the layer deposition was generated from the areas in which there was a concentration of the stresses; in particular, this always happened between one layer and the subsequent one. This is particularly evident especially in cases where the imposed deformation was not parallel to the direction of the fibers (load cases to evaluate *G*_13_ and *G*_23_).

### 3.1. Consideration about the RVE Dimension

**Figure 18:**
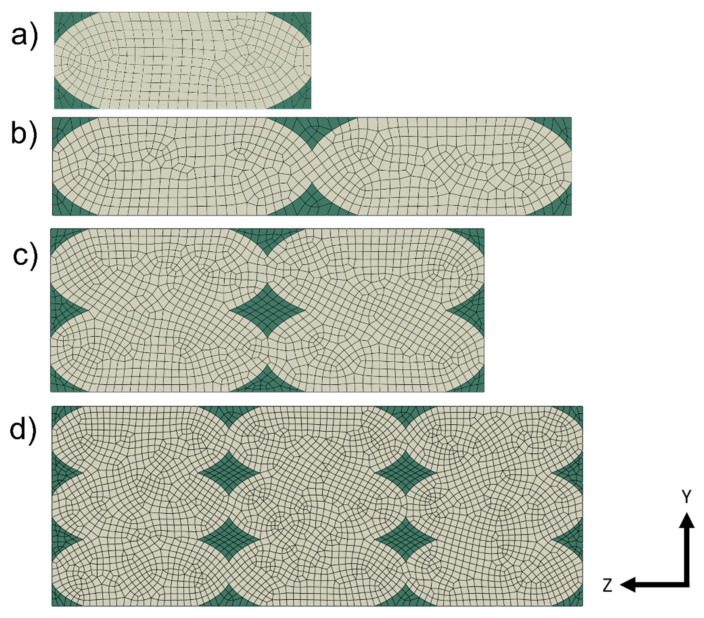
Different RVE dimensions given by number of printed lines used to evaluate the effect of the size influence. (**a**) one line;(**b**) two lines; (**c**) four lines;(**d**) nine lines.

Different RVE dimensions were considered, as shown in Figure 18, to prove that for a linear model with parallel lines the dimension of RVE can be as small as a single line. Moreover, for a widen understanding, the results of the Young’s and Shear moduli of all the models considered were basically the same of the ones reported in Table 3 and Table 4. The variation between the results was less than the 1% and could be related to the discretization of the model. This verified that additional information was not given for a linear model with a linear elastic material by enlarging the RVE dimension. Moreover, when the problem is not linear, the choice of the RVE dimension has a great influence on the modules value, as reported by Okereke and Akpoyomare [32,33]. For this reason, it was decided to use a larger unit cell, which could lead into further research that develops this model.

## 4. Conclusions

The analysis carried out involved the construction of a linear RVE model in order to predict the macroscopic behavior of 3D printed geometries. Particular attention was paid to the geometry of the printed mesostructure observed under a microscope after printing. The given reference material for the model was PLA, which is low-cost and easy to print through FDM methodologies, but with discrete performing mechanical characteristics compared to other thermoplastic polymers. As the similarity between the structure of a 3D FDM-printed component and a UD composite has allowed evaluating the behavior of an FDM-printed sample through a linear elastic RVE.

Subsequently, the construction of a linear elastic model allows obtaining valid results only in the field of small deformations. The condition of the good adhesion between one layer and another is an ideal assumption and constitutes a reference point in the study of these microstructures. The model presented this condition by defining the intersection areas between adjacent lines as a continuous, perfect bonding behavior. This guaranteed a perfect match with the theoretical model and simplified the numerical model.

Furthermore, studies of a complex model in which an adhesion condition is implemented between various printed lines are desirable in order to accurately evaluate intralayer phenomena that are the main cause of failure. The analysis presented showed how the mesoscopic description of the geometry influenced the macroscopic properties of the material, effectively inducing a geometric anisotropy to be considered for the construction of complex components. Future developments are aimed at validating the macroscopic behaviors of the parameters calculated with reference to experimental tests.

## 5. Future Developments

Future developments will consider nonlinear elastic–plastic behaviors common to most polymers to develop a valid model over a wider range of applications. Considerations will also be made about the adhesion between one layer and the next and adjacent layers and its effect on the properties of an RVE. In addition, an experimental setting should be planned to demonstrate the effectiveness of this model.

## Figures and Tables

**Table 1:** Slicing parameters.

Slicing Parameter	Value
Layer height	0.15
Extrusion multiplier	100%
Line width	0.4 mm
Cooling	100
Print temperature	205 °C
Bed temperature	65 °C
Default printing speed	60 mm/s
Line direction	90° *

* Lines were printed along the X direction, considering the reference system reported in both Figure 1 and Figure 2.

**Table 2:** Material properties.

Material	Young’s Modulus (MPa)	Shear Modulus (MPa)	Poisson’s Ratio
PLA	3000	740	0.35
Air	1 × 10^–6^	-	0

**Table 3:** Homogenized elastic properties.

Property	Value	Unit
*E*_11_	2729	MPa
*ν*_12_	0.35	/
*ν*_13_	0.35	/
*E*_22_	2193	MPa
*ν*_21_	0.28	/
*ν*_23_	0.29	/
*E*_33_	2259	MPa
*ν*_31_	0.29	/
*ν*_32_	0.3	/
*G*_12_	870	MPa
*G*_13_	897	MPa
*G*_23_	790	MPa

**Table 4:** Mechanical properties for all the RVE sizes.

	a	b	c	d	Unit
*E*_11_	2729	2729	2729	2729	MPa
*ν*_12_	0.35	0.35	0.35	0.35	/
*ν*_13_	0.35	0.35	0.35	0.35	/
*E*_22_	2196	2194	2193	2195	MPa
*ν*_21_	0.28	0.28	0.28	0.28	/
*ν*_23_	0.29	0.29	0.29	0.29	/
*E*_33_	2261	2261	2259	2257	MPa
*ν*_31_	0.29	0.29	0.29	0.29	/
*ν*_32_	0.30	0.30	0.3	0.29	/
*G*_12_	871	871	870	871	MPa
*G*_13_	897	896	897	896	MPa
*G*_23_	791	791	790	791	MPa

## Data Availability

Not applicable.
